# A Novel Approach for Characterizing Solutions of Rough Optimization Problems Based on Boundary Region

**DOI:** 10.1155/2022/8662289

**Published:** 2022-03-24

**Authors:** Hamiden Abd El- Wahed Khalifa, Dragan Pamucar, Amina Hadj Kacem, W. A. Afifi

**Affiliations:** ^1^Department of Operations Research, Faculty of Graduate Studies for Statistical Research, Cairo University, Giza 12613, Egypt; ^2^Department of Mathematics, College of Science and Arts, Qassim University, Al- Badaya, Saudi Arabia; ^3^Department of Logistics, University of Defence in Belgrade, Belgrade 192204, Serbia; ^4^Mathematics and Statistics Department, College of Science, Taibah University, Yanbu, Saudi Arabia; ^5^Department of Mathematics, Faculty of Science, Tanta University, Tanta, Egypt

## Abstract

Rough set theory, presented by Pawlak in 1981, is one of the most well-known methods for communicating ambiguity by estimating an item based on some knowledge rather than membership. The concept of a rough function and its convexity and differentiability in regard to its boundary region are discussed in this work. The boundary notion is also used to present a new form of rough programming issue and its solutions. Finally, numerical examples are provided to demonstrate the proposed method and emphasize its advantages over other approaches.

## 1. Introduction

Rough set theory has been used to a wide range of problems. In rough set theory, knowledge is said to be dependent on the ability to classify objects, and the indiscernibility relation, which is an equivalence relation, is used to represent it formally [1]. The indiscernibility relation generates an approximation space made up of indiscernible item equivalence classes that spans the entire universe. Pawlak et al. [2] established the concept of a rough set, and one of the most prominent theories to explain ambiguity using the boundary area of a set rather than membership is Pawlak's theory [3]. A rough set, on the one hand, is distinct from ordinary and fuzzy sets in terms of concept. A characteristic function identifies an object in an ordinary set; however, in a fuzzy set, the data's uncertainty is reflected by a partial degree of membership between 0 and 1 [4]. A rough set, on the other hand, approximates an object based on some prior knowledge. The following are some examples of rough mathematical programming problems:

- *1st class:* using a rough feasible set and a crisp objective function and solving mathematical programming problems
- *2nd class*: problems in mathematical programming with a crisp feasible set and a rough target function
- *3rd class:* problems requiring a rough feasible set and a rough objective function in mathematical programming

In rough mathematical programming problems, the ideal solution set is characterized in a rough sense by four optimal sets, each spanning a distinct level of feasibility and optimality [5] (Table 1).

1. FOss is the set of solutions that are certain to be feasible and certain to be optimal
2. FOsp signifies the set of all possible optimal solutions that are certain to be practicable
3. FOps is a term that refers to a collection of all potentially viable and unquestionably optimal alternatives
4. FOpp signifies the collection of all possible and optimal solutions

The viable region in the second class is a crisp set; therefore, FOps = FOss and FOpp = FOsp.

It is clear that FOss ⊆ FOsp ⊂ FOpp, FOss ⊂ FOps ⊂ FOpp, and FOss = FOsp ∩ FOps.

Pawlak et al. [2] and Pawlak [3] were the first to propose the concept of a rough set [3]. Only in these instances, rough set theory is used to represent unclear data, and we just contribute to the “postprocessing step” of the data mining process. Rough multiple objective programming (RMOP) problems are the name for these innovative tasks, and they are grouped into three groups based on where the problem's roughness appears. There are many applications for the rough set theory such as artificial intelligence, expert systems, civil engineering [6–10], medical data analysis [11], data mining (Munakata; [12, 13], Pattern recognition [14]; and [15], and decision theory [16] and [15,17–20], and [21–23]. After turning the random rough variables in the constraint set into crisp ones, Xu and Yao [24] suggested an interactive technique to solve a class of multiobjective programming problems with random rough coefficients. Osman et al. [25] investigated a method for solving a multiobjective transportation issue with rough parameters using a solution approach. Attaya [26] described and solved various objective programming problems with a degree of vagueness in their formulation. Brikaa et al. [27] solved constrained matrix games with fuzzy rough payoff matrices using an effective fuzzy multiobjective programming method. In their proposed model, Ghosh and Roy [28] built a multiobjective product mixing fixed-charge transportation problem with truckload constraints, and an extra cost that was considered as a type-I fixed charge was explored, as well as truck load limits. In a neutrosophic context, Ahmad et al. [29] proposed a new approach for addressing multilevel linear fractional programming problems, with the objective function coefficients represented by rough intervals.

The concept of a rough function, and its convexity and differentiability depending on its boundary region, which are are all important concepts to understand, is introduced in this study. In addition, using the concept of a border region, a novel sort of rough programming challenge is investigated, as well as its answers. Many authors studied the roughness in the optimization problems [30–34].

In terms of its boundary region, this research explores the concept of a rough function, as well as its convexity and differentiability, using inspiration from the above literature. Moreover, the boundary notion is also used to present a new form of rough programming issue and its solutions. It has the distinction due to the inclusion of the following feature time in literature:

1. Rough multiobjective programming problem
2. Kuhn–Tucker. Saddle point of rough programming (RP) problem
3. Optimal solution-based scenario

The following are the study's key goals:

- To distinguish between many forms of optimal solutions for a rough multiobjective programming issue.
- To use a numerical example to validate the suggested study

**Figure 1:**
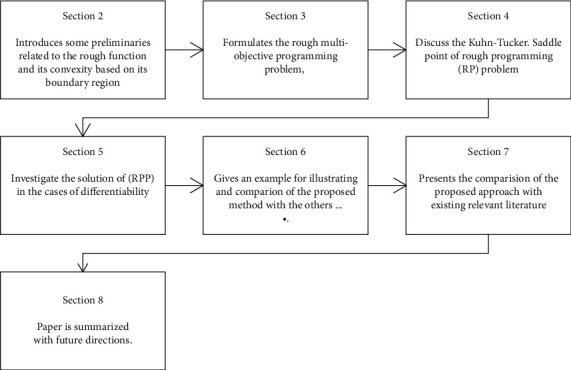
Layout of the remaining paper.

The rest of the paper can be summarized as shown in Figure 1.

## 2. Preliminaries

Some rough function definitions and convexity based on its boundary region are recalled in this part.

**Definition 1 .**

(see [5]). In the rough mathematical programming problem, the optimum value of the objective function is a rough number *f*^*∗*^ specified by lower and upper approximation bounds, denoted by *f*^*∗*(UAI)^ and *f*^*∗*(LAI)^; respectively.

If *f*^*∗*(UAI)^=*f*^*∗*(LAI)^, then the optimal value *f*^*∗*^ is exact, otherwise, *f*^*∗*^ is rough.

Roughness can be found everywhere in the rough mathematical programming problem. Rough feasibility and rough optimality are two novel concepts that have piqued our interest. Only in the first and third classes, where the feasible set is a rough set, rough feasibility does arises. The following solutions have varying degrees of feasibility:

- If a solution *x* ∈ *X* belongs to the lower approximation of the feasible set, it is certain to be feasible
- If a solution *x* ∈ *X* belongs to the upper approximation of the feasible set, it is possibly feasible
- If a solution *x* ∈ *X* does not belong to the upper approximation of the feasible set, it is most likely not feasible
- Rough optimality can be found in a variety of rough mathematical programming problems, with variable degrees of optimality, as demonstrated below:
- if *f*(*x*)=*f*^*∗*(UAI)^, the solution *x* ∈ *X* is unquestionably optimal
- if *f*(*x*) ≥ *f*^*∗*(UAI)^, a solution is possibly optimal
- if (*x*) < *f*^*∗*(UAI)^, a solution *x* ∈ *X* is unquestionably not optimal

In rough mathematical programming problems, the optimal solution sets are defined in a rough sense by four optimal sets covering the different possible degree of feasibility and optimality.

**Definition 2 .**

Let ${\tilde{f}}^{R}:{\mathbb{R}}^{n}⟶\mathbb{R}$ and $r,\overset{˘}{r},r<\overset{˘}{r}$; we suppose that a set of functions *U*(*U*={*f*(*x*), *f*(*x*) : ℝ^*n*^⟶ℝ}) is called the universe set. The set of functions {*f*_*j*_} ⊂ *U* is a lower approximation $L\left({\overset{˜}{f}}^{R}\left(x\right)\right)$ of ${\tilde{f}}^{R}\left(x\right)$ which is denoted by *f*^*LAI*^(*x*) and is defined by $f^{LAI}\left(x\right)=\left\{f_{j}\left(x\right)\in U:\left|f_{j}-{\tilde{f}}^{R}\right|<r\right\}$, and the set of functions {*f*_*k*_} ⊂ *U* is an upper approximation $U\left({\tilde{f}}^{R}\left(x\right)\right)$ of ${\tilde{f}}^{R}\left(x\right)$ which is symbolized by *f*^*UAI*^(*x*) and is characterized with $f^{UAI}\left(x\right)=\left\{f_{k}\left(x\right)\in U:\left|f_{k}-{\tilde{f}}^{R}\right|<\bar{r}\right\}$, where {*f*^LAI^(*x*)} ⊂ {*f*^UAI^(*x*)}. The function ${\tilde{f}}^{R}\left(x\right)$ is called rough function if *f*^LAI^(*x*) ≠ *f*^UAI^(*x*).

**Definition 3 .**

The boundary of the rough function ${\tilde{f}}^{R}\left(x\right)$ is *F*(*x*)=*f*^UAI^(*x*) − *f*^LAI^(*x*), where *f*^*LAI*^(*x*) and *f*^*UAI*^(*x*) are the lower and upper approximation of ${\tilde{f}}^{R}\left(x\right)$.

**Definition 4 .**

Let ${\tilde{f}}^{R}:{\mathbb{R}}^{n}⟶\mathbb{R}$ and $u,\hat{u}\in \mathbb{R},u<\hat{u}$. We suppose that the universal set *V*(*V*={*f*(*x*) : *f* : *ℝ*^*n*^⟶*ℝ*}). The set of functions {*f*_*i*_} ⊂ *V* is the lower and upper approximation of ${\tilde{f}}^{R}$ which is denoted by *f*^LAI^(*x*) and *f*^*UA*^(*x*), respectively, and they are defined as

(1) $$\begin{array}{c} f^{\text{LAI}}\left(x\right)=\left\{f\in V:\left|f_{j}-{\tilde{f}}^{R}\right|<u\right\}, \end{array}$$

(2) $$\begin{array}{c} f^{\text{UAI}}\left(x\right)=\left\{f\in V:\left|f_{j}-{\tilde{f}}^{R}\right|<\hat{u}\right\}. \end{array}$$

The function ${\tilde{f}}^{R}$ is called rough function if

(3) $$\begin{array}{c} f^{\text{LAI}}\left(x\right)\neq f^{\text{UAI}}\left(x\right). \end{array}$$

**Definition 5 .**

The boundary function of the rough function ${\tilde{f}}^{R}$ is *F*(*x*)=*f*^UAI^(*x*) − *f*^LAI^(*x*), where *f*^LAI^ and *f*^UAI^ are defined in (1) and (2), respectively.

**Definition 6 .**

A rough function ${\tilde{f}}^{R}$ is said to be convex if the boundary function *F*(*x*) is convex.

**Definition 7 .**

Let *X* be an open set on *X*. An interval-valued function *f* : *X*⟶*ℝ* with *f*(*x*)=[*f*^LAI^(*x*), *f*^UAI^(*x*)] is called weakly differentiable at *x*_0_ ∈ *X* if the real-valued functions *f*^LAI^ and *f*^UAI^ are differentiable at *x*_0_.

## 3. Problem Statement and Solution Concepts

A rough programming (RP) problem can be stated as

(4) $$\begin{array}{c} \left(RP\right)\min{\tilde{f}}^{R}\left(x\right). \end{array}$$

Subject to

(5) $$\begin{array}{c} X=\left\{x\in {\mathbb{R}}^{n}:h_{r}\left(x\right)\leq 0,r=\bar{1,m}\right\}, \end{array}$$

where *f*_*k*_^LAI^(*x*) and *f*_*k*_^UAI^(*x*) are the lower and upper approximations of ${\tilde{f}}^{R}\left(x\right)$, respectively, and $f_{k}^{\text{LAI}}\left(x\right)\leq {\tilde{f}}_{k}^{R}\left(x\right)\leq f_{k}^{\text{UAI}}\left(x\right),k=\bar{1,K}$, and *X* represents the convex crisp feasible region, and *h*_*r*_(*x*), *r*=1,2,…, *m* are the convex and continuous functions.

In order to solve the (RP) problem, let us solve the following boundary problem (BP):

(6) $$\begin{array}{c} \left(BP\right)\min F\left(x\right)=f^{\text{UAI}}\left(x\right)-f^{\text{LAI}}\left(x\right). \end{array}$$

Subject to

(7) $$\begin{array}{c} X=\left\{x\in {\mathbb{R}}^{n}:h_{r}\left(x\right)\leq 0,r=1,2,\ldots ,m\right\}, \end{array}$$

where *X* is the convex set and *h*_*r*_(*x*), *r*=1,2,…, *m* are the convex and continuous functions.

The BP can be separated into the following two subproblems as

(8) $$\begin{array}{c} \left(\text{LAP}\right)\max f^{\text{LAI}}\left(x\right). \end{array}$$

Subject to

(9) $$\begin{array}{c} X=\left\{x\in {\mathbb{R}}^{n}:h_{r}\left(x\right)\leq 0,r=\bar{1,m}\right\},\\ \left(\text{UAP}\right)\min f^{\text{UAI}}\left(x\right). \end{array}$$

Subject to

(10) $$\begin{array}{c} X=\left\{x\in {\mathbb{R}}^{n}:h_{r}\left(x\right)\leq 0,r=1,2,\ldots ,m\right\}, \end{array}$$

where *f*^LAI^(*x*) and *f*^UAI^(*x*) are the concave and convex functions, respectively.

Let the optimal solutions of (LAP)  and (UAP) be denoted by $f^{\text{LAI}}\left(x^{*}\right)=\underset{x\in X}{\max}f^{\text{LAI}}\left(x\right)$, and

(11) $$\begin{array}{c} f^{\text{UAI}}\left(x^{*}\right)=\underset{x\in X}{\min}f^{\text{UAI}}\left(x\right), \end{array}$$

respectively.

**Definition 8 .**

*x* ^*∗*^ is said to be the optimal solution of the RP problem if $f^{\text{LAI}}\left(x^{*}\right)\leq {\tilde{f}}^{R}\left(x^{*}\right)\leq f^{\text{UAI}}\left(x^{*}\right)$ where *S*^*L*^ and *S*^*U*^ are the sets of the solutions of problems (LAP) and (UAP), respectively.

**Definition 9 .**

1. A solution *x*^*∗*^ ∈ *S*^*L*^∩*S*^*U*^, *F*(*x*^*∗*^)=0 is called a surely optimal solution of the RP
2. *x* ^*∗*^ ∈ *S*^*L*^∩*S*^*U*^, *F*(*x*^*∗*^) ≠ 0 is called a possibly optimal solution of the RP
3. *x* ^*∗*^ ∈ *S*^*L*^∩*S*^*U*^ is called a nearly possibly optimal solution of the RP

**Lemma 1 .**

If *x*^*∗*^ is the solution of (BP), then *x*^*∗*^ is the solution for (LAP) and (UAP).

**Proof**

Let *x*^*∗*^ be a solution of BP; then,

(12) $$\begin{array}{c} f^{\text{UAI}}\left(x^{*}\right)-f^{\text{LAI}}\left(x^{*}\right)\leq f^{\text{UAI}}\left(x\right)-f^{\text{LAI}}\left(x\right);\forall x. \end{array}$$

We suppose that *x*^*∗*^ is not a solution for (LAP) and (UAP), then there exists an $\bar{x}\in X$ such that $f^{\text{UAI}}\left(\bar{x}\right)\leq f^{\text{UAI}}\left(x^{*}\right)$ implies that

$f^{\text{UAI}}\left(\bar{x}\right)-f^{\text{LAI}}\left(\bar{x}\right)<f^{\text{UAI}}\left(x^{*}\right)-f^{\text{LAI}}\left(\bar{x}\right),f^{\text{LAI}}\left(x^{*}\right)<f^{\text{LAI}}\left(\bar{x}\right)$ which leads to

(13) $$\begin{array}{c} f^{\text{UAI}}\left(x^{*}\right)-f^{\text{LAI}}\left(x^{*}\right)>f^{\text{UAI}}\left(x^{*}\right)-f^{\text{LAI}}\left(\bar{x}\right). \end{array}$$

Thus, $f^{\text{UAI}}\left(\bar{x}\right)-f^{\text{LAI}}\left(\bar{x}\right)<f^{\text{UAI}}\left(x^{*}\right)-f^{\text{LAI}}\left(x^{*}\right)$ contradicts that *x*^*∗*^ is a solution of BP. Therefore, *x*^*∗*^ is a solution of the two problems (LAP) and (UAP).

## 4. Rough Kuhn–Tucker Saddle Point

We consider the rough problem

(14) $$\begin{array}{c} \min{\tilde{f}}^{R}\left(x\right). \end{array}$$

Subject to

(15) $$\begin{array}{c} X=\left\{x\in {\mathbb{R}}^{n}:h_{r}\left(x\right)\leq 0,r=\bar{1,m}\right\},\\ f^{\text{LAI}}\left(x\right)\leq {\tilde{f}}^{R}\left(x\right)\leq f^{\text{UAI}}\left(x\right). \end{array}$$

The rough Kuhn–Tucker saddle point for problem (15) takes the form

(16) $$\begin{array}{c} {\tilde{f}}^{R}\left(x^{*}\right)+\overset{m}{\underset{r=1}{\sum }}\gamma_{r}h_{r}\left(x^{*}\right)+\gamma_{m+1}\left(f^{\text{LAI}}\left(x^{*}\right)-{\tilde{f}}^{R}\left(x^{*}\right)\right)+\gamma_{m+2}\left({\tilde{f}}^{R}\left(x^{*}\right)-f^{\text{UAI}}\left(x^{*}\right)\right),\\ \leq {\tilde{f}}^{R}\left(x^{*}\right)+\overset{m}{\underset{r=1}{\sum }}\gamma_{r}^{*}h_{r}\left(x^{*}\right)+\gamma_{m+1}^{*}\left(f^{\text{LAI}}\left(x^{*}\right)-{\tilde{f}}^{R}\left(x^{*}\right)\right)+\gamma_{m+2}^{*}\left({\tilde{f}}^{R}\left(x^{*}\right)-f^{\text{UAI}}\left(x^{*}\right)\right),\\ \leq {\tilde{f}}^{R}\left(x\right)+\overset{m}{\underset{r=1}{\sum }}\gamma_{r}^{*}h_{r}\left(x\right)+\gamma_{m+1}^{*}\left(f^{\text{LAI}}\left(x\right)-{\tilde{f}}^{R}\left(x\right)\right)+\gamma_{m+2}^{*}\left({\tilde{f}}^{R}\left(x\right)-f^{\text{UAI}}\left(x\right)\right), \end{array}$$

or

(17) $$\begin{array}{c} \left(1-\gamma_{m+1}+\gamma_{m+2}\right){\tilde{f}}^{R}\left(x^{*}\right)+\overset{m}{\underset{r=1}{\sum }}\gamma_{r}h_{r}\left(x^{*}\right)+\gamma_{m+1}f^{\text{LAI}}\left(x^{*}\right)-\gamma_{m+2}f^{\text{UAI}}\left(x^{*}\right),\\ \leq \left(1-\gamma_{m+1}^{*}+\gamma_{m+2}^{*}\right){\tilde{f}}^{R}\left(x\right)+\overset{m}{\underset{r=1}{\sum }}\gamma_{r}^{*}h_{r}\left(x^{*}\right)+\gamma_{m+1}^{*}f^{\text{LAI}}\left(x^{*}\right)-\gamma_{m+2}f^{\text{UAI}}\left(x^{*}\right),\\ \leq \left(1-\gamma_{m+1}^{*}+\gamma_{m+2}^{*}\right){\tilde{f}}^{R}\left(x\right)+\overset{m}{\underset{r=1}{\sum }}\gamma_{r}^{*}h_{r}\left(x\right)+\gamma_{m+1}^{*}f^{\text{LAI}}\left(x\right)-\gamma_{m+2}f^{\text{UAI}}\left(x\right). \end{array}$$

**Theorem 1 .**

If (*x*^*∗*^, *γ*_*r*_^*∗*^), where $\gamma_{r}^{*}\geq 0,r=\bar{1,m+2},$ and ∑_*r*=1_^*m*+1^*γ*_*r*_^*∗*^ is a rough Kuhn–Tucker saddle point (KTSP), then *x*^*∗*^ is a solution of RP.

**Proof**

We assume that $\left(x^{*},\gamma_{r}^{*}\right),r=\bar{1,m+2}$ is a rough KTSP; then, for *γ*_*r*_ ≥ 0, *γ*_*r*_ ∈ *ℝ*^*m*+2^, we get

(18) $$\begin{array}{c} \left(1-\gamma_{m+1}+\gamma_{m+2}\right){\tilde{f}}^{R}\left(x^{*}\right)+\overset{m}{\underset{r=1}{\sum }}\gamma_{r}h_{r}\left(x^{*}\right)+\gamma_{m+1}f^{\text{LAI}}\left(x^{*}\right)-\gamma_{m+2}f^{\text{UAI}}\left(x^{*}\right),\\ \leq \left(1-\gamma_{m+1}^{*}+\gamma_{m+2}^{*}\right){\tilde{f}}^{R}\left(x^{*}\right)+\overset{m}{\underset{r=1}{\sum }}\gamma_{r}^{*}h_{r}\left(x^{*}\right)+\gamma_{m+1}^{*}f^{\text{LAI}}\left(x^{*}\right)-\gamma_{m+2}^{*}f^{\text{UAI}}\left(x^{*}\right),\\ \leq \left(1-\gamma_{m+1}^{*}+\gamma_{m+2}^{*}\right){\tilde{f}}^{R}\left(x\right)+\overset{m}{\underset{r=1}{\sum }}\gamma_{r}^{*}h_{r}\left(x\right)+\gamma_{m+1}^{*}f^{\text{LAI}}\left(x\right)-\gamma_{m+2}^{*}f^{\text{UAI}}\left(x\right). \end{array}$$

From the first inequality, we have

(19) $$\begin{array}{c} \left(1-\gamma_{m+1}+\gamma_{m+2}\right){\tilde{f}}^{R}\left(x^{*}\right)+\overset{m}{\underset{r=1}{\sum }}\gamma_{r}h_{r}\left(x^{*}\right)+\gamma_{m+1}f^{\text{LAI}}\left(x^{*}\right)-\gamma_{m+2}f^{\text{UAI}}\left(x^{*}\right),\\ \leq \left(1-\gamma_{m+1}^{*}+\gamma_{m+2}^{*}\right){\tilde{f}}^{R}\left(x\right)+\overset{m}{\underset{r=1}{\sum }}\gamma_{r}^{*}h_{r}\left(x^{*}\right)+\gamma_{m+1}^{*}f^{\text{LAI}}\left(x^{*}\right)-\gamma_{m+2}f^{\text{UAI}}\left(x^{*}\right), \end{array}$$

or

(20) $$\begin{array}{c} \left(1-\gamma_{m+1}+\gamma_{m+2}+1-\gamma_{m+1}^{*}+\gamma_{m+2}^{*}\right){\tilde{f}}^{R}\left(x^{*}\right)+\overset{m}{\underset{r=1}{\sum }}\left(\gamma_{r}-\gamma_{r}^{*}\right)h_{r}\left(x^{*}\right)\\ +\left(\gamma_{m+1}-\gamma_{m+1}^{*}\right)f^{\text{LAI}}\left(x^{*}\right)-\left(\gamma_{m+2}-\gamma_{m+2}^{*}\right)f^{\text{UAI}}\left(x^{*}\right)\leq 0, \end{array}$$

which implies to

(21) $$\begin{array}{c} \left(\gamma_{m+1}-\gamma_{m+1}^{*}\right)\left(f^{\text{LAI}}\left(x^{*}\right)-{\tilde{f}}^{R}\left(x^{*}\right)\right)+\left(\gamma_{m+2}-\gamma_{m+2}^{*}\right)\left({\tilde{f}}^{R}\left(x^{*}\right)-f^{\text{UAI}}\left(x^{*}\right)\right)+\overset{m}{\underset{r=1}{\sum }}\left(\gamma_{r}-\gamma_{r}^{*}\right)h_{r}\left(x^{*}\right)\leq 0. \end{array}$$

This inequality is true for all *γ*_*r*_, *γ*_*r*_^*∗*^, *γ*_*m*+1_, *γ*_*m*+1_^*∗*^, *γ*_*m*+2_, *γ*_*m*+2_^*∗*^. In the case *γ*_*m*+1_=*γ*_*m*+1_^*∗*^ and *γ*_*m*+2_=*γ*_*m*+2_^*∗*^, we have ∑_*r*=1_^*m*^(*γ*_*r*_ − *γ*_*r*_^*∗*^)*h*_*r*_(*x*^*∗*^) ≤ 0. We assume that *γ*_*r*_=*γ*_*r*_^*∗*^, *r*=1,2,…, *i* − 1, *i*+1,…, *m* and *γ*_*i*_^*∗*^=*γ*_*i*_ − 1. Then, *h*_*r*_(*x*^*∗*^) ≤ 0. By repeating this for all *i*, we have *h*_*r*_(*x*^*∗*^) ≤ 0, and hence, x^*∗*^ is the feasible point. Since *γ*_*r*_^*∗*^ ≥ 0 and *h*_*r*_(*x*^*∗*^) ≤ 0, we get ∑_*r*=1_^*m*^*γ*_*r*_^*∗*^*h*_*r*_(*x*^*∗*^) ≤ 0. Again from the first inequality, where *γ*_*m*+1_=*γ*_*m*+1_^*∗*^ and *γ*_*m*+2_=*γ*_*m*+2_^*∗*^, and by setting *γ*_*r*_, we obtain ∑_*r*=1_^*m*^*γ*_*r*_^*∗*^*h*_*r*_(*x*^*∗*^) ≥ 0. Hence, ∑_*r*=1_^*m*^*γ*_*r*_^*∗*^*h*_*r*_(*x*^*∗*^)=0. Thus,

(22) $$\begin{array}{c} \left(\gamma_{m+1}-\gamma_{m+1}^{*}\right)\left(f^{\text{LAI}}\left(x^{*}\right)-{\tilde{f}}^{R}\left(x^{*}\right)\right)+\left(\gamma_{m+2}-\gamma_{m+2}^{*}\right)\left({\tilde{f}}^{R}\left(x^{*}\right)-f^{\text{UAI}}\left(x^{*}\right)\right)+\overset{m}{\underset{r=1}{\sum }}\left(\gamma_{r}-\gamma_{r}^{*}\right)h_{r}\left(x^{*}\right)\leq 0. \end{array}$$

By taking *γ*_*m*+1_=*γ*_*m*+1_^*∗*^ − 1 and *γ*_*m*+2_=*γ*_*m*+2_^*∗*^ − 1, we have $\left(\gamma_{m+1}-1-\gamma_{m+1}^{*}\right)\left(f^{\text{LAI}}\left(x^{*}\right)-{\tilde{f}}^{R}\left(x^{*}\right)\right)+\left(\gamma_{m+2}-1-\gamma_{m+2}^{*}\right)\left({\tilde{f}}^{R}\left(x^{*}\right)-f^{\text{UAI}}\left(x^{*}\right)\right)+\sum_{r=1}^{m}\gamma_{r}h_{r}\left(x^{*}\right)\leq 0.$ This leads to

(23) $$\begin{array}{c} \left(f^{\text{LAI}}\left(x^{*}\right)-{\tilde{f}}^{R}\left(x^{*}\right)\right)+\left({\tilde{f}}^{R}\left(x^{*}\right)-f^{\text{UAI}}\left(x^{*}\right)\right)+\overset{m}{\underset{r=1}{\sum }}\gamma_{r}h_{r}\left(x^{*}\right)\leq 0. \end{array}$$

Since the inequality is valid for each *γ*_*r*_ ≥ 0, then for *γ*_*r*_=0, we get

(24) $$\begin{array}{c} \left(f^{LA}\left(x^{*}\right)-{\tilde{f}}^{R}\left(x^{*}\right)\right)+\left({\tilde{f}}^{R}\left(x^{*}\right)-f^{\text{UAI}}\left(x^{*}\right)\right)\leq 0,\\ f^{\text{UAI}}\left(x^{*}\right)-f^{\text{LAI}}\left(x^{*}\right)\leq 0. \end{array}$$

Taking *γ*_*m*+1_=*γ*_*m*+1_^*∗*^+1 and *γ*_*m*+2_=*γ*_*m*+2_^*∗*^+1, we have

(25) $$\begin{array}{c} \left(\gamma_{m+1}+1-\gamma_{m+1}^{*}\right)\left(f^{\text{LAI}}\left(x^{*}\right)-{\tilde{f}}^{R}\left(x^{*}\right)\right)+\left(\gamma_{m+2}+1-\gamma_{m+2}^{*}\right)\left({\tilde{f}}^{R}\left(x^{*}\right)-f^{\text{UAI}}\left(x^{*}\right)\right)+\overset{m}{\underset{r=1}{\sum }}\gamma_{r}h_{r}\left(x^{*}\right)\leq 0. \end{array}$$

Thus,

(26) $$\begin{array}{c} \left(f^{\text{LAI}}\left(x^{*}\right)-{\tilde{f}}^{R}\left(x^{*}\right)\right)+\left({\tilde{f}}^{R}\left(x^{*}\right)-f^{\text{UAI}}\left(x^{*}\right)\right)+\overset{m}{\underset{r=1}{\sum }}\gamma_{r}h_{r}\left(x^{*}\right)\leq 0. \end{array}$$

Since the inequality is valid for each *γ*_*r*_ ≥ 0, then for *γ*_*r*_=0, we have

(27) $$\begin{array}{c} \left(f^{\text{LAI}}\left(x^{*}\right)-{\tilde{f}}^{R}\left(x^{*}\right)\right)+\left({\tilde{f}}^{R}\left(x^{*}\right)-f^{\text{UAI}}\left(x^{*}\right)\right)\leq 0,\\ f^{\text{UAI}}\left(x^{*}\right)-f^{\text{LAI}}\left(x^{*}\right)\geq 0. \end{array}$$

Hence from (24) and (27), we conclude $f^{\text{LAI}}\left(x^{*}\right)={\tilde{f}}^{R}\left(x^{*}\right)=f^{\text{UAI}}\left(x^{*}\right)$ (i.e., *x*^*∗*^ is a surely optimal solution for the RP)

From the second inequality, we have

(28) $$\begin{array}{c} \left(1-\gamma_{m+1}^{*}+\gamma_{m+2}^{*}\right){\tilde{f}}^{R}\left(x^{*}\right)+\overset{m}{\underset{r=1}{\sum }}\gamma_{r}^{*}h_{r}\left(x^{*}\right)+\gamma_{m+1}^{*}f^{LA}\left(x^{*}\right)-\gamma_{m+2}f^{\text{UAI}}\left(x^{*}\right),\\ \leq \left(1-\gamma_{m+1}^{*}+\gamma_{m+2}^{*}\right){\tilde{f}}^{R}\left(x\right)+\overset{m}{\underset{r=1}{\sum }}\gamma_{r}^{*}h_{r}\left(x\right)+\gamma_{m+1}^{*}f^{\text{LAI}}\left(x\right)-\gamma_{m+2}^{*}f^{\text{UAI}}\left(x\right). \end{array}$$

Since ∑_*r*=1_^*m*^*γ*_*r*_^*∗*^*h*_*r*_(*x*^*∗*^)=0, then

(29) $$\begin{array}{c} \left(1-\gamma_{m+1}^{*}+\gamma_{m+2}^{*}\right)\left({\tilde{f}}^{R}\left(x^{*}\right)-{\tilde{f}}^{R}\left(x\right)\right)\leq \overset{m}{\underset{r=1}{\sum }}\gamma_{r}^{*}h_{r}\left(x\right)+\gamma_{m+1}^{*}\left(f^{\text{LAI}}\left(x\right)-f^{\text{LAI}}\left(x^{*}\right)\right)\\ +\gamma_{m+2}^{*}\left(f^{\text{UAI}}\left(x\right)-f^{\text{UAI}}\left(x^{*}\right)\right){\tilde{f}}^{R}\left(x^{*}\right)-{\tilde{f}}^{R}\left(x\right)\leq \frac{\sum_{r=1}^{m}\gamma_{r}^{*}}{\left(1-\gamma_{m+1}^{*}+\gamma_{m+2}^{*}\right)}h_{r}\left(x\right)\\ +\frac{\gamma_{m+1}^{*}}{\left(1-\gamma_{m+1}^{*}+\gamma_{m+2}^{*}\right)}\left(f^{\text{LAI}}\left(x\right)-f^{\text{LAI}}\left(x^{*}\right)\right)+\frac{\gamma_{m+2}^{*}}{\left(1-\gamma_{m+1}^{*}+\gamma_{m+2}^{*}\right)}\left(f^{\text{UAI}}\left(x\right)-f^{\text{UAI}}\left(x^{*}\right)\right). \end{array}$$

For *x*^*∗*^ ∈ *S*^*L*^∩*S*^*U*^, we have *f*^LAI^(*x*) ≤ *f*^LAI^(*x*^*∗*^) and *f*^UAI^(*x*) ≥ *f*^UAI^(*x*^*∗*^). Since ∑_*r*=1_^*m*+1^*γ*_*r*_=1 and *γ*_*m*+1_^*∗*^=*γ*_1_^*∗*^+*γ*_1_^*∗*^+⋯+*γ*_*m*_^*∗*^, then 1 − *γ*_*m*+1_^*∗*^+*γ*_*m*+2_^*∗*^ ≤ 0 which implies to ${\tilde{f}}^{R}\left(x^{*}\right)\leq {\tilde{f}}^{R}\left(x\right),x\in X$. Hence, *x*^*∗*^ is a possible optimalsolution of the rough problem. For *x*^*∗*^ ∈ *S*^*L*^, *x*^*∗*^ ∉ *S*^*U*^, we obtain *f*^LAI^(*x*^*∗*^)≥*f*^LAI^(*x*) and

(30) $$\begin{array}{c} {\tilde{f}}^{R}\left(x^{*}\right)-{\tilde{f}}^{R}\left(x\right)\leq \frac{\gamma_{m+2}^{*}}{\left(1-\gamma_{m+1}^{*}+\gamma_{m+2}^{*}\right)}\left(f^{\text{UAI}}\left(x\right)-f^{\text{UAI}}\left(x^{*}\right)\right). \end{array}$$

Now, there are two cases:

**Case 1 .**

*f* ^UAI^(*x*^*∗*^) − *f*^UAI^(*x*) ≤ 0; ∀*x* ∈ *X* implies that x^*∗*^ is a nearly possibly optimal solution.

**Case 2 .**

*f* ^*UAI*^(*x*^*∗*^) − *f*^*UAI*^(*x*) > 0.

Let x^*∗*^ be not a nearly possible optimal solution of rough problem; then, there is $\bar{x}\in X:{\tilde{f}}^{R}\left(\bar{x}\right)<{\tilde{f}}^{R}\left(x^{*}\right)$. Since *x*^*∗*^ ∈ *S*^*L*^, *x*^*∗*^ ∉ *S*^*U*^, so *x*^*∗*^ is not a solution for the boundary problem BP, i.e., there is $\bar{x}:$

(31) $$\begin{array}{c} f^{\text{UAI}}\left(\bar{x}\right)-f^{\text{LAI}}\left(\bar{x}\right)<f^{\text{UAI}}\left(x^{*}\right)-f^{\text{LAI}}\left(x^{*}\right),f^{\text{LAI}}\left(x^{*}\right)-f^{\text{LAI}}\left(\bar{x}\right)<f^{\text{UAI}}\left(x^{*}\right)-f^{\text{UAI}}\left(\bar{x}\right). \end{array}$$

- If $f^{\text{UAI}}\left(x^{*}\right)<f^{\text{UAI}}\left(\bar{x}\right)$, then $f^{\text{LAI}}\left(x^{*}\right)<f^{\text{LAI}}\left(\bar{x}\right)$. This contradicts that *x*^*∗*^ ∈ *S*^*L*^, and hence, *x*^*∗*^ must be a nearly possible optimal solution for the RP problem.
- If $f^{\text{UAI}}\left(x^{*}\right)>f^{\text{UAI}}\left(\bar{x}\right)$, then we may write $f^{\text{UAI}}\left(x^{*}\right)=f^{\text{UAI}}\left(\bar{x}\right)+\theta ,\theta >0$, which implies to $f^{\text{LAI}}\left(x^{*}\right)-f^{\text{LAI}}\left(\bar{x}\right)<\theta ,\theta >0$. Then, we have two cases:

- $f^{\text{LAI}}\left(x^{*}\right)>f^{\text{LAI}}\left(\bar{x}\right)$, which is not considered, where *x*^*∗*^ ∈ *S*^*L*^
- $f^{\text{LAI}}\left(x^{*}\right)<f^{\text{LAI}}\left(\bar{x}\right)$, which contradicts that *x*^*∗*^ ∈ *S*^*L*^, and hence, *x*^*∗*^ must be a nearly possible optimal solution for the RP problem

For *x*^*∗*^ ∈ *S*^*U*^, *x*^*∗*^ ∉ *S*^*U*^, we obtain *f*^UAI^(*x*^*∗*^)≤*f*^UAI^(*x*) and

(32) $$\begin{array}{c} {\tilde{f}}^{R}\left(x^{*}\right)-{\tilde{f}}^{R}\left(x\right)\leq \frac{\gamma_{m+1}^{*}}{\left(1-\gamma_{m+1}^{*}+\gamma_{m+2}^{*}\right)}\left(f^{\text{LAI}}\left(x\right)-f^{\text{LAI}}\left(x^{*}\right)\right). \end{array}$$

So, there are two cases:

**Case 3 .**

*f* ^LAI^(*x*^*∗*^) − *f*^LAI^(*x*) ≤ 0; ∀*x* ∈ *X*; this implies that *x*^*∗*^ is a nearly possibly optimal solution.

**Case 4 .**

*f* ^LAI^(*x*^*∗*^) − *f*^LAI^(*x*) > 0.

Let *x*^*∗*^ be not a nearly possible optimal solution of the rough problem; then, there is $\bar{x}\in X:{\tilde{f}}^{R}\left(\bar{x}\right)<{\tilde{f}}^{R}\left(x^{*}\right)$. Since *x*^*∗*^ ∈ *S*^*U*^, *x*^*∗*^ ∉ *S*^*L*^, so x^*∗*^ is not a solution for the boundary problem (BP), i.e., there is $\bar{x}\in X:$

(33) $$\begin{array}{c} f^{\text{UAI}}\left(\bar{x}\right)-f^{\text{LAI}}\left(\bar{x}\right)<f^{\text{UAI}}\left(x^{*}\right)-f^{\text{LAI}}\left(x^{*}\right),f^{\text{UAI}}\left(\bar{x}\right)-f^{\text{UAI}}\left(x^{*}\right)<f^{\text{LAI}}\left(\bar{x}\right)-f^{\text{UA}}\left(x^{*}\right). \end{array}$$

- If $f^{\text{LAI}}\left(\bar{x}\right)<f^{\text{UAI}}\left(x^{*}\right)$, then $f^{\text{UAI}}\left(\bar{x}\right)<f^{\text{LAI}}\left(x^{*}\right)$. This contradicts that *x*^*∗*^ ∈ *S*^*U*^, and hence, *x*^*∗*^ must be a nearly possible optimal solution for the (R − MOP) problem.
- If $f^{\text{LAI}}\left(\bar{x}\right)>f^{\text{UAI}}\left(x^{*}\right)$, then we may write $f^{\text{LAI}}\left(x^{*}\right)=f^{\text{LAI}}\left(\bar{x}\right)+\theta ,\theta >0$, which implies to $f^{\text{UAI}}\left(\bar{x}\right)-f^{\text{UAI}}\left(\bar{x}\right)<\theta ,\theta >0$ Then, we have two cases:

- *f*^LAI^(*x*^*∗*^) > *f*^LAI^(*x*^*∗*^), which is not considered, where *x*^*∗*^ ∈ *S*^*U*^
- $f^{\text{UAI}}\left(x^{*}\right)<f^{\text{UAI}}\left(\bar{x}\right)$, which contradicts that *x*^*∗*^ ∈ *S*^*U*^, and hence, x^*∗*^ must be a nearly possible optimal solution for the RP problem

## 5. Rough Function Differentiability

A rough function ${\tilde{\text{f}}}^{\text{R}}\left(x\right)$ is said to be differentiable if its boundary.

*F*(*x*)=*f*^UAI^ − *f*^LAI^ is differentiable. Then,

*F* − *F*(*x*^*∗*^)=(*δ*/*δx*)*F*(*x*^*∗*^)(*x* − *x*^*∗*^)+*ϑ*(*x*^*∗*^, *γ*(*x* − *x*^*∗*^))‖*x* − *x*^*∗*^‖, or equivalently

${\tilde{f}}^{R}-{\tilde{f}}^{R}\left(x^{*}\right)=\left(\delta /\delta x\right){\tilde{f}}^{R}\left(x^{*}\right)\left(x-x^{*}\right)+\vartheta \left(x^{*},\gamma \left(x-x^{*}\right)\right)\left\|x-x^{*}\right\|$ , where

(34) $$\begin{array}{c} \underset{\vartheta ⟶0}{\lim}\vartheta \left(x^{*},\delta \left(x-x^{*}\right)\right)=0. \end{array}$$

### 5.1. Kuhn–Tucker's Conditions under Roughness

The rough Kuhn–Tucker (KT) conditions for the RP problem takes the form

(35) $$\begin{array}{c} \frac{\delta }{\delta x}{\tilde{f}}^{R}\left(x^{*}\right)+\overset{m}{\underset{r=1}{\sum }}\gamma_{r}^{*}h_{r}\left(x^{*}\right)+\gamma_{m+1}^{*}\frac{\delta }{\delta x}\left(f^{\text{LAI}}\left(x^{*}\right)-{\tilde{f}}^{R}\left(x^{*}\right)\right)+\gamma_{m+2}^{*}\frac{\delta }{\delta x}\left({\tilde{f}}^{R}\left(x^{*}\right)-f^{\text{UAI}}\left(x^{*}\right)\right)=0,\\ \gamma_{r}^{*}h_{r}\left(x^{*}\right)=0,r=\bar{1,m;}\gamma_{m+2}^{*}\left({\tilde{f}}^{R}\left(x^{*}\right)-f^{\text{UAI}}\left(x^{*}\right)\right)=0;\gamma_{r}^{*}\geq 0,r=\bar{1,m+2}. \end{array}$$

Let ∑_*r*=1_^*m*+1^*γ*_*r*_^*∗*^=1. Then,

(36) $$\begin{array}{c} \left(1-\gamma_{m+1}^{*}+\gamma_{m+2}^{*}\right)\frac{\delta }{\delta x}{\tilde{f}}^{R}\left(x^{*}\right)+\gamma_{m+1}^{*}\frac{\delta }{\delta x}f^{\text{LAI}}\left(x^{*}\right)-\gamma_{m+2}^{*}\frac{\delta }{\delta x}f^{\text{UAI}}\left(x^{*}\right)+\overset{m}{\underset{r=1}{\sum }}\gamma_{r}^{*}\frac{\delta }{\delta x}h_{r}\left(x^{*}\right)=0, \end{array}$$

or, in other words

(37) $$\begin{array}{c} \frac{\delta }{\delta x}{\tilde{f}}^{R}\left(x^{*}\right)+\frac{\gamma_{m+1}^{*}}{\left(1-\gamma_{m+1}^{*}+\gamma_{m+2}^{*}\right)}\frac{\delta }{\delta x}f^{\text{LAI}}\left(x^{*}\right)-\frac{\gamma_{m+2}^{*}}{\left(1-\gamma_{m+1}^{*}+\gamma_{m+2}^{*}\right)}\frac{\delta }{\delta x}f^{\text{UAI}}\left(x^{*}\right)\\ +\frac{\sum_{r=1}^{m}\gamma_{r}^{*}}{\left(1-\gamma_{m+1}^{*}+\gamma_{m+2}^{*}\right)}\frac{\delta }{\delta x}h_{r}\left(x^{*}\right)=0\\ \frac{\sum_{r=1}^{m}\gamma_{r}^{*}}{\left(1-\gamma_{m+1}^{*}+\gamma_{m+2}^{*}\right)}\frac{\delta }{\delta x}h_{r}\left(x^{*}\right)=0,\\ r=\bar{1,m},\\ \frac{\gamma_{m+1}^{*}}{\left(1-\gamma_{m+1}^{*}+\gamma_{m+2}^{*}\right)}f^{\text{LAI}}\left(x^{*}\right)=0\\ \frac{\gamma_{m+2}^{*}}{\left(1-\gamma_{m+1}^{*}+\gamma_{m+2}^{*}\right)}f^{\text{UAI}}\left(x^{*}\right)\\ \gamma_{r}^{*}\geq 0,\\ r=\bar{1,m+2}. \end{array}$$

**Theorem 2 .**

Let ${\tilde{f}}^{R},f^{\text{UAI}}$, and *h* be the convex and differentiable functions at *x*^*∗*^, and let f^LAI^ be a concave and differentiable at *x*^*∗*^ ∈ *X*. We suppose that f^UAI^(*x*^*∗*^) >0 and *f*^LAI^(*x*^*∗*^) > 0. If (*x*^*∗*^, *γ*_*r*_^*∗*^), where $\gamma_{r}^{*}\geq 0,r=\bar{1,m+2}$ is a solution of the KT conditions, then *x*^*∗*^ is a solution for RP

**Proof**

Let (*x*^*∗*^, *γ*_*r*_^*∗*^) be a solution of the rough KT conditions. Since ${\tilde{\text{f}}}^{\text{R}}$ is a convex and differentiable at *x*^*∗*^, we get ${\tilde{f}}^{R}-{\tilde{f}}^{R}\left(x^{*}\right)\geq \delta x/\delta {\tilde{f}}^{R}\left(x^{*}\right)\left(x-x^{*}\right)$. Since $\delta /\delta x{\tilde{f}}^{R}\left(x^{*}\right)=\gamma_{m+2}^{*}/\left(1-\gamma_{m+1}^{*}+\gamma_{m+2}^{*}\right)\delta /\delta xf^{UAI}\left(x^{*}\right)-\gamma_{m+1}^{*}/\left(1-\gamma_{m+1}^{*}+\gamma_{m+2}^{*}\right)\delta /\delta xf^{LAI}\left(x^{*}\right)-\sum_{r=1}^{m}\gamma_{r}^{*}/\left(1-\gamma_{m+1}^{*}+\gamma_{m+2}^{*}\right)h_{r}\left(x^{*}\right)$, and *f*^*UA*^, *f*^*LA*^, and h_r_ are differentiable, then

(38) $$\begin{array}{c} f^{\text{UAI}}-f^{\text{UAI}}\left(x^{*}\right)=\frac{\delta }{\delta x}f^{\text{UAI}}\left(x^{*}\right)\left(x-x^{*}\right)+\vartheta \left(x^{*},\gamma \left(x-x^{*}\right)\right)x-x^{*},\\ f^{\text{LAI}}-f^{\text{LAI}}\left(x^{*}\right)=\frac{\delta }{\delta x}f^{LA}\left(x^{*}\right)\left(x-x^{*}\right)+\vartheta \left(x^{*},\gamma \left(x-x^{*}\right)\right)x-x^{*},\\ h_{r}-h_{r}\left(x^{*}\right)=\frac{\delta }{\delta x}h_{r}\left(x^{*}\right)\left(x-x^{*}\right)+\vartheta \left(x^{*},\gamma \left(x-x^{*}\right)\right)x-x^{*}. \end{array}$$

Then,

(39) $$\begin{array}{c} {\tilde{f}}^{R}-{\tilde{f}}^{R}\left(x^{*}\right)\geq \frac{\gamma_{m+2}^{*}}{\left(1-\gamma_{m+1}^{*}+\gamma_{m+2}^{*}\right)}\left(f^{UA}-f^{UA}\left(x^{*}\right)-\vartheta \left(x^{*},\gamma \left(x-x^{*}\right)\right)x-x^{*}\right)\\ -\frac{\gamma_{m+1}^{*}}{\left(1-\gamma_{m+1}^{*}+\gamma_{m+2}^{*}\right)}\left(f^{\text{LAI}}-f^{\text{LAI}}\left(x^{*}\right)-\vartheta \left(x^{*},\gamma \left(x-x^{*}\right)\right)x-x^{*}\right)\\ -\frac{\sum_{r=1}^{m}\gamma_{r}^{*}}{\left(1-\gamma_{m+1}^{*}+\gamma_{m+2}^{*}\right)}\left(h_{r}-h_{r}\left(x^{*}\right)-\vartheta \left(x^{*},\gamma \left(x-x^{*}\right)\right)x-x^{*}\right). \end{array}$$

Since lim_*ϑ*⟶0_*ϑ*(*x*^*∗*^, *δ*(*x* − *x*^*∗*^))=0, then

(40) $$\begin{array}{c} {\tilde{f}}^{R}-{\tilde{f}}^{R}\left(x^{*}\right)\geq \frac{\gamma_{m+2}^{*}}{\left(1-\gamma_{m+1}^{*}+\gamma_{m+2}^{*}\right)}\left(f^{\text{UAI}}-f^{\text{UAI}}\left(x^{*}\right)\right)-\frac{\gamma_{m+1}^{*}}{\left(1-\gamma_{m+1}^{*}+\gamma_{m+2}^{*}\right)}\left(f^{\text{LAI}}-f^{\text{LAI}}\left(x^{*}\right)\right)\\ -\frac{\sum_{r=1}^{m}\gamma_{r}^{*}}{\left(1-\gamma_{m+1}^{*}+\gamma_{m+2}^{*}\right)}\left(h_{r}-h_{r}\left(x^{*}\right)\right). \end{array}$$

From the Kuhn–Tucker conditions,

(41) $$\begin{array}{c} \frac{\sum_{r=1}^{m}\gamma_{r}^{*}}{\left(1-\gamma_{m+1}^{*}+\gamma_{m+2}^{*}\right)}h_{r}\left(x^{*}\right)=0,\\ r=\bar{1,m},\\ \frac{\gamma_{m+1}^{*}}{\left(1-\gamma_{m+1}^{*}+\gamma_{m+2}^{*}\right)}f^{\text{LAI}}\left(x^{*}\right)=0,\\ \frac{\gamma_{m+2}^{*}}{\left(1-\gamma_{m+1}^{*}+\gamma_{m+2}^{*}\right)}f^{\text{UAI}}\left(x^{*}\right)=0. \end{array}$$

Then, the following inequality ${\tilde{f}}^{R}\left(x\right)-{\tilde{f}}^{R}\left(x^{*}\right)\geq \gamma_{m+2}^{*}/\left(1-\gamma_{m+1}^{*}+\gamma_{m+2}^{*}\right)f^{UAI}-\gamma_{m+1}^{*}/\left(1-\gamma_{m+1}^{*}+\gamma_{m+2}^{*}\right)f^{LAI}-\sum_{r=1}^{m}\gamma_{r}^{*}/\left(1-\gamma_{m+1}^{*}+\gamma_{m+2}^{*}\right)h_{r}$ is valid for each $\gamma_{r}^{*}\geq 0,r=\bar{1,m+2}$, and for *γ*_*r*_^*∗*^=0, we have

(42) $$\begin{array}{c} {\tilde{f}}^{R}-{\tilde{f}}^{R}\left(x^{*}\right)\geq \frac{\gamma_{m+2}^{*}}{\left(1-\gamma_{m+1}^{*}+\gamma_{m+2}^{*}\right)}f^{\text{UAI}}-\frac{\gamma_{m+1}^{*}}{\left(1-\gamma_{m+1}^{*}+\gamma_{m+2}^{*}\right)}f^{\text{LAI}}. \end{array}$$

If *γ*_*m*+1_^*∗*^; *γ*_*m*+2_^*∗*^ > 0, then from the Kuhn–Tucker conditions, we obtain ${\tilde{f}}^{R}\left(x^{*}\right)=f^{\text{LAI}}\left(x^{*}\right)$ and ${\tilde{f}}^{R}\left(x^{*}\right)=f^{\text{UAI}}\left(x^{*}\right)$. Then, *x*^*∗*^ is a surely optimal solution of the RP problem.

If *x*^*∗*^ ∈ *S*^*L*^∩*S*^*U*^, then *f*^UAI^(*x*^*∗*^) ≤ *f*^UAI^(*x*); ∀*x* ∈ *X* and *f*^LAI^(*x*^*∗*^) ≥ *f*^LAI^(*x*); ∀*x* ∈ *X*, and then we grt

(43) $$\begin{array}{c} {\tilde{f}}^{R}-{\tilde{f}}^{R}\left(x^{*}\right)\geq \frac{\gamma_{m+2}^{*}}{\left(1-\gamma_{m+1}^{*}+\gamma_{m+2}^{*}\right)}f^{\text{UAI}}\left(x^{*}\right)-\frac{\gamma_{m+1}^{*}}{\left(1-\gamma_{m+1}^{*}+\gamma_{m+2}^{*}\right)}f^{\text{LAI}}\left(x^{*}\right). \end{array}$$

In addition, from the Kuhn–Tucker conditions ${\tilde{f}}^{R}-{\tilde{f}}^{R}\left(x^{*}\right)\geq 0$, this leads to ${\tilde{f}}^{R}\left(x^{*}\right)\leq {\tilde{f}}^{R}\left(x\right)$, i.e., x^*∗*^ is a possibly optimal solution.

If *x*^*∗*^ ∈ *S*^*L*^, *x*^*∗*^ ∉ *S*^*U*^, then *f*^LAI^(*x*^*∗*^) ≥ *f*^LAI^(*x*); ∀*x* ∈ *X*, and we have

(44) $$\begin{array}{c} {\tilde{f}}^{R}-{\tilde{f}}^{R}\left(x^{*}\right)\geq \frac{\gamma_{m+2}^{*}}{\left(1-\gamma_{m+1}^{*}+\gamma_{m+2}^{*}\right)}f^{\text{UAI}}\left(x^{*}\right)-\frac{\gamma_{m+1}^{*}}{\left(1-\gamma_{m+1}^{*}+\gamma_{m+2}^{*}\right)}f^{\text{LAI}}\left(x^{*}\right),\\ {\tilde{f}}^{R}-{\tilde{f}}^{R}\left(x^{*}\right)\geq \frac{\gamma_{m+2}^{*}}{\left(1-\gamma_{m+1}^{*}+\gamma_{m+2}^{*}\right)}f^{\text{UAI}}. \end{array}$$

From the assumption that *f*^UAI^(*x*^*∗*^) > 0, and *x*^*∗*^ is not solution for BP, *γ*_*m*+2_^*∗*^/(1 − *γ*_*m*+1_^*∗*^+*γ*_*m*+2_^*∗*^)=0.

Hence, ${\tilde{f}}^{R}-{\tilde{f}}^{R}\left(x^{*}\right)\geq 0$ leads to ${\tilde{f}}^{R}\left(x^{*}\right)\leq {\tilde{f}}^{R};\forall x$. Then, *x*^*∗*^ is a nearly possibly optimal solution for RP.

If *x*^*∗*^ ∈ *S*^*U*^, *x*^*∗*^ ∉ *S*^*L*^; then, *f*^UAI^(*x*^*∗*^) ≤ *f*^UAI^(*x*); ∀*x*, and we have

(45) $$\begin{array}{c} {\tilde{f}}^{R}\left(x\right)-{\tilde{f}}^{R}\left(x^{*}\right)\geq \frac{\gamma_{m+2}^{*}}{\left(1-\gamma_{m+1}^{*}+\gamma_{m+2}^{*}\right)}f^{\text{UAI}}\left(x^{*}\right)-\frac{\gamma_{m+1}^{*}}{\left(1-\gamma_{m+1}^{*}+\gamma_{m+2}^{*}\right)}f^{\text{LAI}}\left(x^{*}\right). \end{array}$$

From KT conditions, we have

(46) $$\begin{array}{c} {\tilde{f}}^{R}-{\tilde{f}}^{R}\left(x^{*}\right)\geq \frac{\gamma_{m+1}^{*}}{\left(1-\gamma_{m+1}^{*}+\gamma_{m+2}^{*}\right)}f^{\text{LAI}}. \end{array}$$

From the assumption that *f*^LAI^(*x*^*∗*^) > 0, and *x*^*∗*^ is not solution for BP,

(47) $$\begin{array}{c} \frac{\gamma_{m+1}^{*}}{\left(1-\gamma_{m+1}^{*}+\gamma_{m+2}^{*}\right)}=0. \end{array}$$

Thus, ${\tilde{f}}^{R}\left(x\right)-{\tilde{f}}^{R}\left(x^{*}\right)\geq 0$, which implies to ${\tilde{f}}^{R}\left(x^{*}\right)\leq {\tilde{f}}^{R}\left(x\right);\forall x$. Then, *x*^*∗*^ is a nearly possibly optimal solution for RP.

## 6. Numerical Example

We consider the following problem ${\tilde{f}}^{R}\left(x\right):X⟶\mathbb{R}$ with f^LAI^(*x*)=*x*_1_+*x*_2_*f*^UAI^(*x*)=1/3*x*_1_^3^ − 2*x*_1_^2^ − 10*x*_2_+100 and consider the following RP problem as

(48) $$\begin{array}{c} \left(RP\right)\min{\tilde{f}}^{R}\left(x\right). \end{array}$$

Subject to

(49) $$\begin{array}{c} X=\left\{\left(x_{1},x_{2}\right)\in {\mathbb{R}}^{2}:x_{1}+x_{2}\leq 10,3.5\leq x_{1}\leq 6,x_{2}\leq 6,x_{1}+x_{2}\geq 1\right\}. \end{array}$$

Then,

(50) $$\begin{array}{c} \left(\text{LAP}\right)\min f^{\text{LAI}}\left(x\right)=x_{1}+x_{2}. \end{array}$$

Subject to

(51) $$\begin{array}{c} x\in X,\\ \left(\text{UAP}\right)\min f^{\text{UAI}}\left(x\right)=\frac{1}{3}x_{1}^{3}-2x_{1}^{2}-10x_{2}+100. \end{array}$$

Subject to

(52) $$\begin{array}{c} x\in X. \end{array}$$

Hence, the BP is

(53) $$\begin{array}{c} \left(BP\right)\min F\left(x\right)=f^{\text{UAI}}\left(x\right)-f^{\text{LAI}}\left(x\right). \end{array}$$

Subject to

(54) $$\begin{array}{c} x\in X. \end{array}$$

The solution of the LAP is *S*^*L*^={(5,5)}, and the solution of the UAP is S^*U*^={(1 − *λ*)(6,4)+*λ*(4,6), 0 ≤ *λ* ≤ 1}. Then,

1. There is no one-size-fits-all answer (Definition 9.1)
2. The best conceivable solution is (5,5), where (5,5) ∈ *S*^*L*^∩*S*^*U*^ and F(5,5) ≠ 0 (Definition 9.2)
3. The nearly possibly solution is {(1 − *λ*)(6,4)+*λ*(4,6), 0 ≤ *λ* ≤ 1} ∪ {(5,5)} (Definition 9.3)

## 7. Discussion

The proposed approach is compared to some existing literature in this section to show the benefits of the proposed approach.  Table 2 investigates this comparison in the case of some parameters

## 8. Concluding Remarks and Future Works

This paper introduces the concept of a rough function, as well as its convexity and differentiability based on its boundary region. The boundary area notion has also been used to investigate a new sort of rough programming challenge and its answers. This research could be expanded to include more fuzzy-like structures in the future (such as interval-valued fuzzy sets, neutrosophic sets, pythagorean fuzzy sets, and spherical fuzzy sets), and more discussion and suggestions could also be included in the future studies. The key features of this study can be summarized as follows:

The proposed study can be extended by developing

- Intuitionistic fuzzy set with a possibility interval value
- Intuitionistic fuzzy set with a probability interval
- Fuzzy hypersoft expert set is a possibility
- Possibility fuzzy pythagorean set
- Possibility picture fuzzy set
- For example, a spherical fuzzy set

The following are some ideas for further research:

1. For rough multiobjective programming, determine the link between rough weights and rough parameters
2. An investigation of duality in the context of a rudimentary multiobjective programming problem
3. A parametric study of a rough programming issue in which the objective function has roughness
4. A parametric investigation of a rough programming problem with rough constraints
5. Determine the link between the rough weights and the rough parameter in rough multiobjective programming
6. A duality investigation on the problem of rough multiobjective programming
7. A parametric analysis of a rough programming problem in which the goal function and restrictions are both rough [36–38]

## Figures and Tables

**Table 1:** Optimal solution set covering the different possible degree of feasibility and optimality.

		Optimality	
		Possibly	Surely
Feasibility	Possibly	FOpp	FOps
	Surely	FOsp	FOss

**Table 2:** Comparisons of different researchers' contributions.

Author's name	Weighting method	*ε*− constraint method	KKT optimality	Efficient solution	Parametric study	Environment
Khalifa [30]	**√**	**×**	**×**	**√**	**×**	Rough set
Osman et al. [25]	**×**	**×**	**×**	**√**	**×**	Fuzzy set
Ammar and Emsimir [31]	**√**	**×**	**√**	**√**	**×**	Fuzzy set
Ahmed [35]	**×**	**×**	**√**	**√**	**×**	Fuzzy set
Ammar and Al- Al- Asfar [32]	**√**	**×**	**√**	**√**	**×**	Fuzzy set
Our investigation	**√**	**√**	**√**	**√**	**√**	Rough set

## Data Availability

No data were used in this manuscript.
